# Superficial and deep white matter abnormalities in temporal lobe epilepsy

**DOI:** 10.1093/braincomms/fcaf305

**Published:** 2025-08-19

**Authors:** Gerard R Hall, Sarah J Gascoigne, Jonathan J Horsley, Yujiang Wang, Csaba Kozma, Jane de Tisi, Sjoerd B Vos, Gavin P Winston, John S Duncan, Peter N Taylor

**Affiliations:** CNNP Lab (www.cnnp-lab.com), School of Computing, Newcastle University, Newcastle upon Tyne NE4 5BX, United Kingdom; CNNP Lab (www.cnnp-lab.com), School of Computing, Newcastle University, Newcastle upon Tyne NE4 5BX, United Kingdom; CNNP Lab (www.cnnp-lab.com), School of Computing, Newcastle University, Newcastle upon Tyne NE4 5BX, United Kingdom; CNNP Lab (www.cnnp-lab.com), School of Computing, Newcastle University, Newcastle upon Tyne NE4 5BX, United Kingdom; Translational and Clinical Research Institute, Faculty of Medical Sciences, Newcastle University, Newcastle upon Tyne, UK; Department of Clinical and Experimental Epilepsy, UCL Queen Square Institute of Neurology, University College London, London, UK; CNNP Lab (www.cnnp-lab.com), School of Computing, Newcastle University, Newcastle upon Tyne NE4 5BX, United Kingdom; Department of Clinical and Experimental Epilepsy, UCL Queen Square Institute of Neurology, University College London, London, UK; Department of Clinical and Experimental Epilepsy, UCL Queen Square Institute of Neurology, University College London, London, UK; Western Australia National Imaging Facility Node, The University of Western Australia, Nedlands, Australia; Centre for Medical Image Computing, Computer Science Department, University College London, London, UK; Department of Clinical and Experimental Epilepsy, UCL Queen Square Institute of Neurology, University College London, London, UK; Division of Neurology, Department of Medicine, Queen’s University, Kingston, Canada; Department of Clinical and Experimental Epilepsy, UCL Queen Square Institute of Neurology, University College London, London, UK; CNNP Lab (www.cnnp-lab.com), School of Computing, Newcastle University, Newcastle upon Tyne NE4 5BX, United Kingdom; Translational and Clinical Research Institute, Faculty of Medical Sciences, Newcastle University, Newcastle upon Tyne, UK; Department of Clinical and Experimental Epilepsy, UCL Queen Square Institute of Neurology, University College London, London, UK

**Keywords:** temporal lobe epilepsy, diffusion MRI, diffusion-weighted imaging, fractional anisotropy, white matter abnormality

## Abstract

Non-invasive neuroimaging is important in epilepsy to help identify cerebral abnormalities. Abnormally reduced fractional anisotropy (FA) in deep white matter (WM) from diffusion-weighted imaging (DWI) is widely reported in large multi-cohort studies across all types of epilepsies. However, abnormalities in FA for superficial WM are rarely investigated in epilepsy. To gain a greater understanding of the nature of WM abnormality at different WM depths, we investigated DWI abnormalities at a range of superficial and deep WM in two separate temporal lobe epilepsy (TLE) cohorts. The first cohort (TLE = 81, Healthy Control; HC = 67) underwent a high angular resolution multi-shell DWI, whilst the second cohort (TLE = 70, HC = 29) had a single-shell acquisition. We registered FA maps to a standard template, and analysed temporal WM within 8 mm of the temporal lobe grey matter, amygdala and hippocampus. We standardised FA measures at different depths, and compared ipsi-versus contralateral temporal WM, and MRI-positive versus MRI-negative groups. We report three major findings: First, superficial WM had greater FA reductions than deep WM in TLE (*P* < 0.001). Second, this effect was more prominent in the ipsilateral than contralateral temporal lobe WM (*P* < 0.001). Third, these effects were present to a similar degree in patients who reported an MRI negative. All results are held in both TLE cohorts. These findings suggest that, in the temporal lobe, superficial WM is more abnormal than deep WM in TLE, with potential clinical use for lateralisation even in MRI-negative patients. These findings motivate further investigation of the importance of superficial WM in epilepsy.

## Teaser

Hall *et al*. investigate whether fractional anisotropy abnormality in temporal lobe white matter is spatially homogenous at differing depths in drug-resistant temporal lobe epilepsy. The main findings show that temporal white matter and superficial to cortical and subcortical grey matter are more structurally abnormal than deeper white matter.

## Graphical Abstract

**Graphical Abstract:**
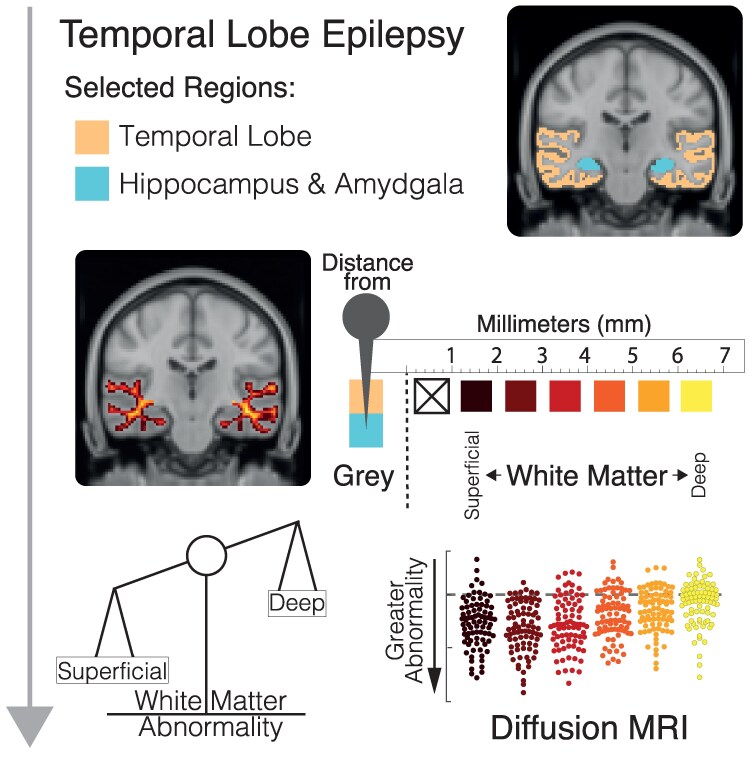

## Introduction

Epilepsy affects over 60 million people,^1^ with a third of cases resistant to anti-seizure medications.^2^ Temporal lobe epilepsy (TLE) is the most common form of drug-resistant focal epilepsy.

Structural MRI methods (T1w and T2w-FLAIR) are the most widespread and commonly used neuroimaging methods to investigate changes in TLE. Large multi-cohort studies have reported widespread abnormalities in grey matter measures of cortical thickness and subcortical volumes, particularly in the ipsilateral hippocampal volume.^3^ To complement this information, other MRI techniques can provide additional crucial information. Diffusion-weighted imaging (DWI) is effective for identifying subtle differences in white matter (WM), often quantified on a voxel basis. The most widely used model is the diffusion tensor, which describes the tensor shape of the underlying tissue properties using quantitative measures. The fractional anisotropy (FA) of the tensor is widely reported to be reduced in epilepsy, with the strongest effects in TLE, especially in individuals with hippocampal sclerosis.^4-8^

Most DWI research focuses on major tracts located in deep WM, with less attention on WM closer to the cortical surface. This is due to increased partial volume effects from neighbouring grey matter and lower uniformity in tissue morphometry.^9^ Few studies have investigated superficial WM in TLE. Despite these challenges, research has revealed important findings that could improve abnormality detection. Studies have reported reduced FA in both deep and superficial WM, especially near temporolimbic regions, with greater superficial WM abnormalities in individuals with hippocampal atrophy.^10^ Others reported widespread superficial and deep WM abnormalities in TLE with hippocampal sclerosis, more pronounced ipsilaterally.^11,12^ Superficial WM abnormalities have been linked to cognitive phenotypes.^13^ Superficial WM are more likely to reflect local connectivity, including ‘u’ fibers, and deep WM are more likely to reflect long-range association fibers, so it is important that both are considered. Scanning parameters of DWI may also influence abnormality measures, for example, multi-shell techniques sample diffusivity at multiple scales rather than one single scale. Therefore, both single and multi-shell DWI are included.

In the current study, we investigated the nature of superficial and deep WM abnormalities in individuals with drug-resistant TLE. We then compared ipsi- and contralateral temporal WM changes, and investigated MRI-negative patients. Finally, we replicated our findings in an independent cohort and compared ipsi—contralateral temporal WM for lateralization in individuals.

## Materials and methods

### Participants

The first cohort was 81 individuals with drug-resistant TLE and 67 healthy controls. The second cohort comprised 70 individuals with drug-resistant TLE and 29 healthy controls. More information about each cohort is in the appendices, Table 1 and Table 2.

**Table 1:** *Post-hoc* analysis for temporal lobe FA differences between hemispheres for each distance (ipsilateral against contralateral)

WM Distance group	*μ* (mean difference)	Cohens *d*	*μ* Standard error	*P* value	*μ* 95% Confidence interval	
					Lower bound	Upper bound
[1.0–2.0]	−0.865	−0.865	0.10	<0.001	−1.061	−0.669
[2.0–3.0]	−0.884	−0.884	0.100	<0.001	−1.078	−0.690
[3.0–4.1]	−0.752	−0.752	0.094	<0.001	−0.940	−0.565
[4.1–5.1]	−0.579	−0.579	0.100	<0.001	−0.779	−0.379
[5.1–6.1]	−0.340	−0.341	0.120	0.006	−0.578	−0.103
[6.1–7.1]	−0.354	−0.354	0.131	0.009	−0.616	−0.093

All *post-hoc* analyses underwent a Tukey–Kramer for multiple comparison corrections.

**Table 2:** Post-hoc analysis for differences between hemisphere for each distance (ipsilateral against contralateral)

					*μ* 95% Confidence interval	
WM Distance group	*μ* (mean difference)	Cohens *d*	*μ* Standard error	*P* value	Lower bound	Upper bound
[1.0–2.0]	−0.924	−0.872	0.129	<0.001	−1.181	−0.666
[2.0–3.0]	−0.844	−0.861	0.127	<0.001	−1.098	−0.590
[3.0–4.1]	−0.881	−0.893	0.150	<0.001	−1.179	−0.583
[4.1–5.1]	−0.774	−0.785	0.144	<0.001	−1.062	−0.487
[5.1–6.1]	−0.338	−0.338	0.163	0.042*	−0.663	−0.013
[6.1–7.1]	−0.270	−0.270	0.162	0.100	−0.591	0.053

All *post-hoc* analyses were corrected with a Tukey–Kramer for multiple comparison corrections.

### MRI acquisition and pre-processing

#### Cohort 1

The data of this cohort were collected between 2014 and 2019. T1-weighted (T1w) and diffusion-weighted MRI were obtained using a 3T GE MR750 scanner, equipped with a body coil for transmission and a 32-channel phased array coil for reception. Standard imaging gradients with a maximum strength of 50 mtm-1 and slew rate 200 Tm-1s-1 were fitted. Structural T1*_w_* were collected with an inversion- recovery fast spoiled gradient recalled echo (FSPGR) sequence with the following parameters [TE = 3.1 ms, TR = 7.4 ms, inversion time = 400 ms, 166 contiguous, field of view (FOV) = 224 × 256 × 256 mm, matrix = 224 × 256 × 256, voxel size = 1.00 × 1.00 × 1.00 mm = 1.00 mm^3^, parallel imaging acceleration factor = 2]. Diffusion-weighted MRI data were acquired using a single-shot spin-echo planar imaging sequence with echo time = 74.1 ms and repetition time 7600 ms. Sets of 70 contiguous 2-mm-thick axial slices were obtained covering the whole brain. A total of 115 volumes were acquired with 11, 8, 32, and 64 gradient directions at b-values of 0, 300, 700, and 2500 s/mm^2^, respectively (*δ* = 21.5 ms, *Δ* = 35.9 ms) as well as a single B_0_ image with reverse phase-encoding. The field of view was 25.6 × 25.6 cm, and the acquisition matrix size was 128 × 128, giving a reconstructed voxel size of 2 × 2 × 2 mm.

The DWI data were denoised,^14^ corrected for Gibbs ringing artifact^15^ and corrected for signal drift.^16^ We also used TOPUP and EDDY^17^ from the FSL toolbox^18-20^ to correct for motion, and eddy current-induced distortions. We used the Synb0-DISCO tool^21,22^ to generate a synthetic, undistorted b0 from the individual’s corresponding T1w image. Finally, correction for bias fields was applied using ‘dwibiascorrect’ in MRtrix using the ANTs N4biasfield tool.^23^

#### Cohort 2

The data of this cohort were collected at an earlier timepoint, between 2009 and 2013. T1-weighted (T1w) and diffusion-weighted MRI were obtained using a 3T GE Signa HDx scanner equipped with an eight-channel phased array coil. Structural T1w images were collected with an FSPGR acquisition with the following parameters (TE = 3.04 ms, TR = 37.68 s, 170 contiguous, 1.1mm-thick coronal slices containing 256 × 256 matrix, 0.9375 × 0.9375 mm in-plane resolution). Diffusion MRI was collected using a cardiac-triggered single-shot echo planar imaging acquisition [TE = 73 ms, TR = heart-rate dependent, b-value of 1200 s/mm^2^ (*δ* = 21 ms, *Δ* = 29 ms, using maximum gradient strength of 40 mT m−1), 52 directions with 6 b0. Overall, 60 axial slices were collected, each 2.4-mm thick with a 96 × 96 matrix, zero-filled to 128 × 128 giving, 1.875 × 1.875 mm in-plane resolution]. DWI data were pre-processed using an identical method to the first cohort.

### DWI post-processing

All diffusion tensor maps were reconstructed using FSL’s DTIFIT.^18-20^ All b-values were used in tensor reconstruction for both cohorts. Once maps were constructed, the FA map of each participant was registered using ANTs toolbox^23^ to the ‘FMRIB158_2mm’, a FA-based template provided from FSL toolbox. Registration involved both linear (affine) and non-linear registrations (SYN-Diffeomorphic). Then, subsequent linear and non-linear transformations generated from the prior registration were applied to all the tensor metrics (FA, AD, RD and MD) using a trilinear interpolation. Each diffusion map was manually quality checked before and after the processing steps, to check for problems in registration and/or pre-processing.

**Figure 1:**
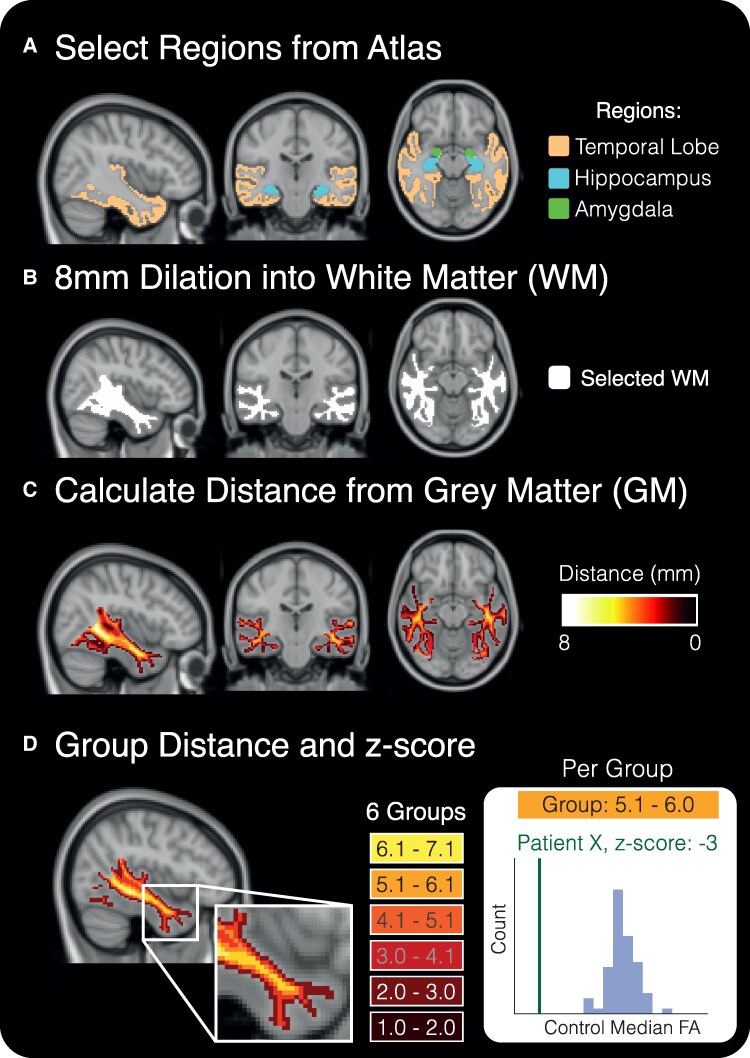
**Methods summary to calculate distance maps and groups.** (**A**) All cortical temporal lobe regions, including the subcortical hippocampus and amygdala were selected from the ‘MNI-152_T1_1mm’ Template. (**B**) We dilated 8 mm into the WM tissue mask from the cortical temporal lobe regions to best capture the underlying temporal lobe WM. Only WM voxel distances and DWI tensor values were captured within this mask. (**C**) Euclidean distance in millimeters (mm) from the closest GM region was calculated for each WM voxel. (**D**) Distances were combined into non-overlapping groups, and control distributions of median FA were calculated per distance group for each of the cohort controls. Age and sex effects were modelled in controls and applied to patients. Individual patient *z*-scores of median FA were calculated per distance from the mean and standard deviation of the controls.

To calculate the template distance map, the ‘MNI-152_T1_1mm’ template was processed in Freesurfer.^24^ Temporal lobe neocortical regions, in addition to hippocampus and amygdala, were selected as regions of interest (ROIs) (Fig. 1, Section A). Only WM voxels within 8 mm of the selected ROIs were analysed (Fig. 1, Section B). Then we used the ‘distancemap’ tool from the FSL toolbox to calculate the Euclidean distance transform between each WM voxel in the mask and their closest distance to the nearest grey matter (GM) voxels selected from our ROIs (Fig. 1, Section C). Since the template was in the same space as the ‘FMRIB158_2mm’ template, all masks were resampled into a 2-mm space. Then the values calculated for the distances were sorted into six different non-overlapping groups between 1.0 and 7.1 mm (that range being the minimum and maximum distance measured), each group approximately 1 mm in size [exact distance groups in mm are as follows: (1–2.0235), (2.0236–3.0470), (3.0471–4.0706), (4.0707–5.0942), (5.0943–6.1178), (6.1179–7.1414)]. We discarded any voxels with <1 mm distance to the GM-WM boundary to reduce partial voluming. Average FA per distance was calculated using the median FA. A step-wise linear regression was then performed on the controls of each cohort to determine age and sex differences in FA at each distance, if significant; corrections were applied to patients and controls by distance, group and hemisphere.

### Statistical testing

Averaged FA for each distance group was calculated for the controls in each cohort. Normality was assessed by inspecting distributions and using a one-sample Kolmogorov-Smirnov test (’kstest’ in MATLAB_2023b). From visual inspection, median FA showed better normality than the mean, so only the median FA was used for patient z-score generation. All patient *z*-scores were calculated based on the hemispheric and distance-specific mean and standard deviation of their respective controls (Fig. 1, section D). At every distance group per hemisphere, all patients were *z*-scored against their respective controls.

We performed a repeated measures ANOVA on the TLE group’s *z*-scores (‘ranova’ in MATLAB_2023b), in which the two within-group factors were the hemisphere (left and right) and distance (6 groups). For the second analysis, an independent *t*-test was used to compare the MRI-positive and negative TLE groups. ‘crosstab’ in MATLAB_2023b was run to analyse the effectiveness of superficial WM on lateralization.

The last analysis compared the strongest z-scores between ipsilateral-contralateral temporal lobe WM, per distance group within each patient. In order to analyze the significance of the proportion of correctly lateralized patients (patients with a stronger ipsilateral abnormality), we ran a one-sample binomial test (‘binocdf’ in MATLAB_2023b) to compare our proportion against random chance (50%).

## Results

### Reduced FA in superficial ipsilateral white matter

**Figure 2:**
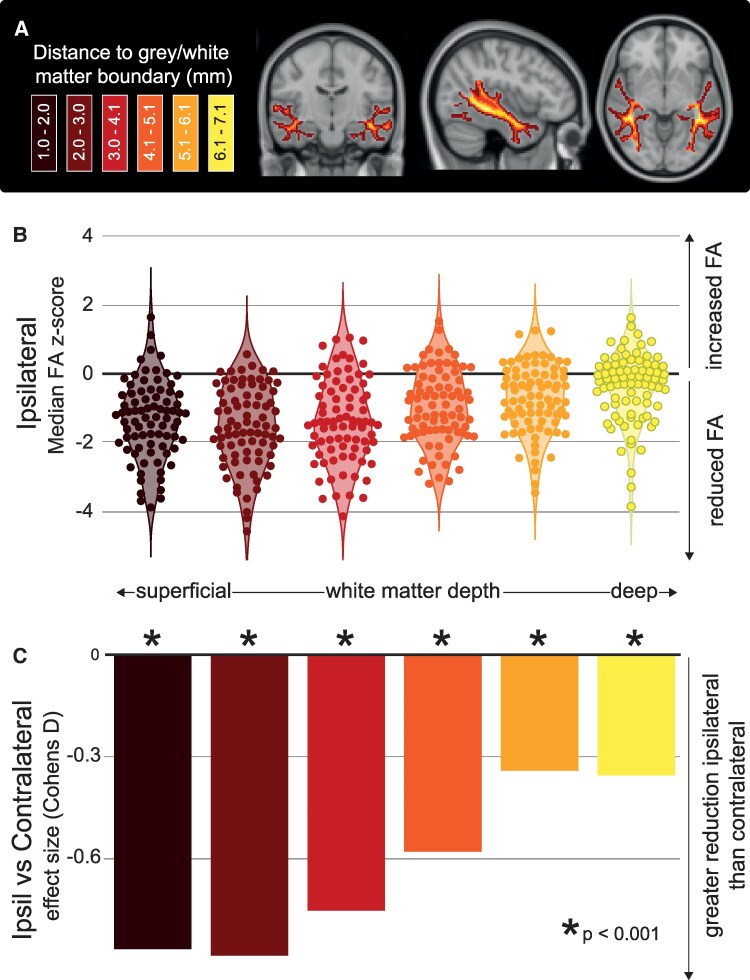
**Relative to distance, reductions in FA are greater at the superficial distances in TLE.** (**A**)(Left) Illustration of the WM distances from the GM boundary separated into six groups. Each group has a unique colour gradient between dark red (Superficial WM) and bright yellow (Deep WM). (**A**)(Right) Each of the six distance groups overlaid on the MNI-152 template for anatomical reference. (**B**) Grouped scatter plot where each datapoint represents the ipsilateral median FA z-score for each individual with TLE (*n* = 81) at all 6 WM distance groups from GM. (**C**) Mean effect size difference (Cohen’s d) of *z*-scored median FA within each Ipsilateral and Contralateral hemisphere for every distance. Significance was reported using a repeated measures ANOVA.

At different WM depths (Fig. 2A), we investigated ipsilateral FA reductions in individuals with TLE (Fig. 2B). In superficial WM (dark data points, left side of Fig. 2B), FA is reduced substantially in patients—each data point represents an individual patient. In deeper WM (lighter data points, right side of Fig. 2B), FA is much less altered in patients. Overall, it is visually apparent that FA reductions are considerably more pronounced in superficial WM than in deep WM.

To statistically quantify if a specific dMRI abnormalities are influenced by WM distance in the ipsilateral temporal lobe in TLE, we conducted a repeated measures ANOVA. The two within-group variables were distance (groups 1–6) and hemisphere (ipsilateral and contralateral to epileptogenic foci). Mauchly’s test reported the assumption of sphericity had been violated, $X^{2}(65)=602.96,P<0.001$, requiring a Greenhouse-Geisser correction to be applied to the following data. We found a significant effect for distance F(5,400)=22.26,P<0.001. Furthermore, the test reported a significant effect within hemisphere F(1,80)=65.84,P<0.001, and the interaction between hemisphere and distance F(5,400)=8.81,P<0.001. See also Supplementary Tables A6, and A7 for further details, along with Figs A2-A4, A6-A7.

Given the significant effect of hemisphere for temporal lobe WM, we compared ipsilateral to contralateral temporal lobe FA per distance by computing the effect size (Cohen’s d). Figure 2C shows the effect size between the hemisphere of each temporal lobe at different WM depths. Negative values indicate greater FA reductions in the temporal lobe ipsilateral to the epileptogenic foci rather than contralateral. Ipsilateral FA reductions are more pronounced in superficial WM.

There were greater reductions of FA in the temporal lobe ipsilateral than contralateral to the epileptogenic foci (μ=−0.63,SE=0.08,P<.001). Additional post-hoc analysis investigating comparisons of the ipsilateral and contralateral temporal lobes at each individual distance is shown in Table 1. In comparison to the contralateral temporal lobe, significant reductions of median FA in the ipsilateral temporal lobe were present at every WM distance group.

Together, the results in Fig. 2 indicate that the FA in temporal lobe WM is (i) most reduced in superficial, rather than deep WM, (ii) more reduced ipsilaterally than contralaterally to the epileptogenic foci, and (iii) the ipsilateral reduction is greater at superficial levels than at contralateral superficial levels.

### Superficial reductions in white matter FA are present even in MRI-negative patients

**Figure 3:**
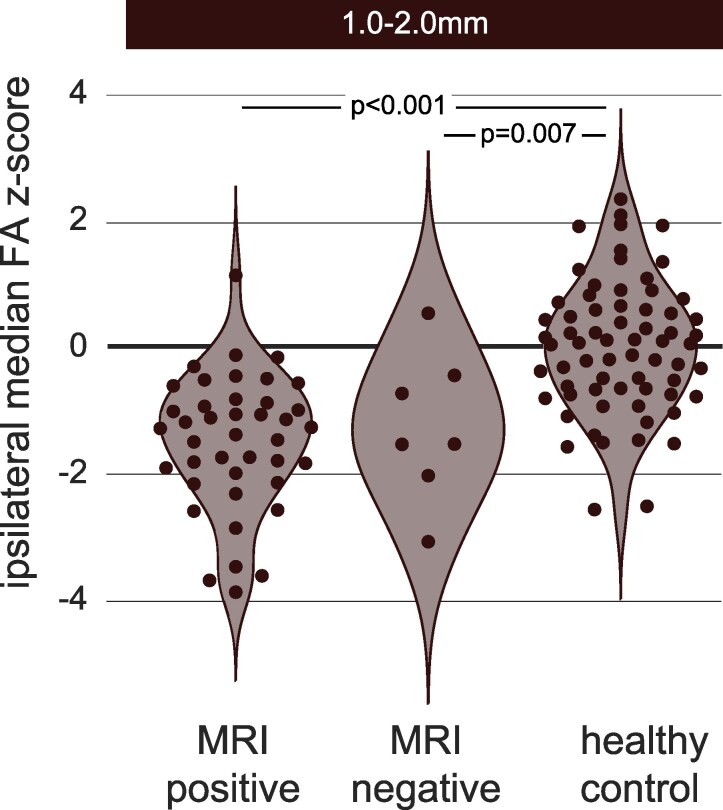
**Similar group abnormalities were present for both MRI-positive HS and MRI-negative individuals against controls at the most superficial WM distances.** Each point represents an individual patient or controls (*n* = 67), ipsilateral median FA *z*-score (average of both hemispheres for controls). There were no significant differences in ipsilateral median FA between the MRI-positive HS (*n* = 74) and MRI-negative individuals (*n* = 7) with TLE. MRI positive: MRI evidence of hippocampal sclerosis, MRI negative: no reported MRI pathology. Significance was calculated using an independent samples *t*-test. WM: white matter.

To investigate whether ipsilateral temporal lobe reductions in the most superficial WM distances were present in MRI negative individuals, we analysed the three most superficial WM groups (1–2 mm, 2–3 mm, 3–4.1 mm) out of the six originally analysed in the prior analysis. Figure 3 shows that the most superficial distance (1–2 mm) was reduced FA in both the MRI-positive (hippocampal sclerosis) and MRI-negative groups, relative to controls (t(106)=−7.27,P<.001, and t(72)=−3.04,P=.007, respectively). This same difference was also present for the following two most superficial WM distances [(2–3mm: MRI-positive: t(106)=−7.97,P<.001; MRI-negative: t(72)=−2.80,P=0.01; Fig. A1), (3–4.1mm: MRI-positive: t(106)=−6.94,P<.001; MRI-negative: t(72)=−2.37,P=0.04; Fig. A1)]. Superficial white matter exhibits reduced FA, in MRI-negative patients. Full information on all statistical comparisons can be found in Supplementary Table A4, and a table listing post-operative pathology of each group is reported in Supplementary Table A3.

### Replication of results in a second TLE cohort with different dMRI parameters

We tested the reproducibility of the previous two results. Therefore, we re-ran the same analysis on cohort 2: an independent cohort of individuals with TLE and controls that underwent dMRI scanning with different parameters at an earlier time point. The second cohort replicated all previous analyses.

**Figure 4:**
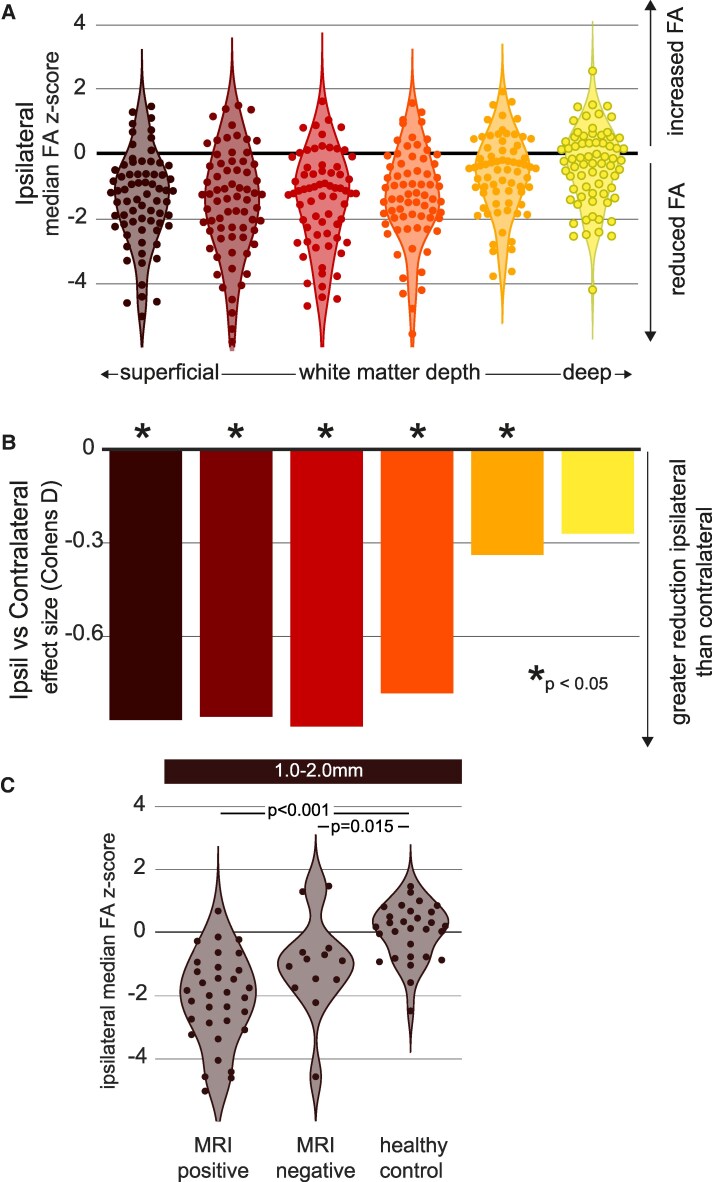
**Results are replicated in a different TLE cohort with changes in scanning parameters, corresponding illustrations to (A) (B) and (C) are Figs 2B, 2C and 3, respectively.** (**A**) Strongest ipsilateral reductions are evident at the superficial distances compared to the deeper distances; each datapoint represents an individual with TLE (*n* = 70) for each distance group. (**B**) When analysing each ipsilateral distance group to its contralateral counterpart, all distances were significantly different except for the deepest distance [6.1–7.1]. Strongest effect sizes were similarly present at 5 mm and below. Significance was reported using a repeated measures ANOVA. (**C**) Both MRI-positive (*n* = 57) and MRI-negative (*n* = 13) groups were significantly different from controls (*n* = 29) at the three most superficial distances. Each datapoint in the group scatterplot represents an individual at the 1.0–2.0 distance group. MRI-positive patients with hippocampal sclerosis, and MRI-negative patients had no visual MRI abnormalities. Statistical significance was calculated using an independent samples *t*-test.

Statistically this involved running a repeated measures ANOVA defining two within-group variables; hemisphere (ipsilateral, contralateral), and distance (distance groups 1–6) of temporal lobe WM. Mauchly’s test again reported the assumption of sphericity had been violated, $X^{2}(65)=573.30,P<.001$; therefore, a Greenhouse–Giesser correction was applied on the following results. All results are displayed in Fig. 4, significant effects for Hemisphere F(1,69)=39.86,P<.001, Distance F(5,345)=18.49,P<.001 , and interaction between Hemisphere and Distance of temporal lobe WM F(5,345)=6.67,P<.001. The results from the repeated measures ANOVA are consistent with the prior cohort (Tables 2, Supplementary Table A5).

Replicating the second analysis, we compared the MRI-positive HS (n=33) and MRI-negative (n=13) groups against controls at three of the most superficial WM distances. After normality checking, the independent samples *t*-test reported significant differences against controls for both MRI-positive HS and MRI-negative HS at all superficial distances. [1.0–2.0 mm]: MRI positive t(60)=−7.03,  P<0.001; MRI negative t(40)=2.83,P=0.015, Fig. 4.C; [2.0–3.0 mm]: MRI positive t(60)=−5.70,P<0.001; MRI negative t(40)=−4.11,P<0.001, Fig. A1; and [3.0–4.1 mm] MRI positive t(60)=−5.50,P<0.001; MRI negative t(40)=5.11,P<0.001, Fig. A1. *P*-values were adjusted with a Bonferoni correction.

### Superficial WM abnormalities can lateralise in TLE

With prior analyses showing strong effect size differences between the ipsilateral and contralateral temporal lobe WM, particularly at the superficial distance groups (Fig. 2.C & 4.C), we next investigated their potential for lateralization.

**Figure 5:**
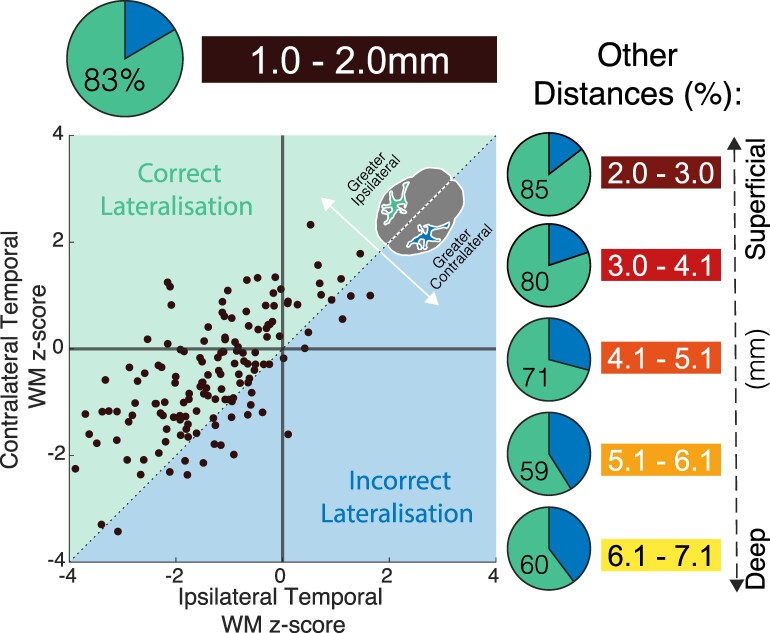
**Superficial WM is accurate at lateralising epileptogenic tissue in TLE.** When comparing ipsilateral against contralateral temporal WM *z*-scores per patient in all cohorts (*n* = 151), the majority of individuals had stronger abnormal ipsilateral *z*-scores. This effect is most prominent at the superficial WM distance groups. (Left) Plot shows each individual patient’s (represented as a dot) ipsilateral WM z-score in relation to their contralateral equivalent for the 1.0–2.0 mm distance group. If the patient dot is in the green, the ipsilateral z-score is greater therefore suggesting correct lateralization. The overall percentage of correct lateralization is represented by the green, and percentage in the pie chart. (Right) Pie chart percentage overviews for the other five distance groups. ‘Correct Lateralization’ meaning the ipsilateral temporal lobe was more abnormal than the contralateral temporal lobe at that distance. A one-sample binomial test was run to calculate significance. WM: white matter.

In Fig. 5, each data point represents an individual with TLE, and y-axis values are identical to those shown in Figs 2B and 4A (i.e. the ipsilateral abnormality). The x-axis values represent the contralateral abnormality. As such, any patient in the green shaded area has greater abnormality ipsilaterally than contralaterally. Some 83% of patients occur in the green area for the most superficial WM (1–2 mm). To assess the significance of this finding (as compare to a random split of 50%) we used a one-sample binomial test. Results were significant at all depths, but more pronounced superficially ([1.0–2.0 mm]: 126/151, 83.4%,P<.001; [2.0–3.0 mm]: 129/151, 85.4%,P<.001; [3.0–4.1 mm]: 121/151, 80.1%,P<.001; [4.1–5.1 mm]: 107/151, 70.9%,P<.001; [5.1–6.1 mm]: 89/151, 58.9%,  P=0.01; [6.1–7.1 mm]: 91/151, 60.3%,P=0.01). Overall, the superficial distance groups showed stronger lateralization than the deep WM distances.

## Discussion

We investigated DWI white matter abnormality at a range of depths from cortical grey matter in the temporal lobe. This approach of investigating multiple depths represents a key novelty of our study. Three major findings were evident in temporal lobe WM; first, TLE generally had stronger FA abnormality in the superficial than deep white matter. Second, reduced superficial FA was particularly evident ipsilateral to the epileptogenic foci. Third, these reductions were evident in patients reported as MRI negative. Our findings were reproduced in a separate TLE cohort, with different DWI acquisition parameters.

### Greater DWI abnormality in superficial than deep temporal lobe WM for TLE

For the TLE group, superficial WM revealed greater abnormality than deeper WM in the temporal lobe. These differences were evident in the ipsilateral hemisphere, with the strongest ipsilateral-contralateral difference in WM 5 mm and closer to the cortical surface for two separate drug-resistant TLE cohorts. This finding is consistent with prior research investigating superficial WM in drug-resistant TLE with hippocampal sclerosis, showing the strongest FA effect size in the temporal lobe (middle and superior temporal gyri).^11^ The same study also reported differences in superficial WM FA in temporolimbic regions, in a drug-resistant TLE group. Furthermore, a link was found between superficial multivariate diffusion scores (consisting of FA and MD) and hippocampal volume, linking superficial WM FA and MD measures to the severity of TLE pathology.^10^ Our findings, combined with previous findings, support micro-structural changes in TLE at the superficial WM are robust, reproducible, and readily detectable using DWI. Recent research has combined *in vivo* DWI tensor measures with histological findings in drug-resistant TLE to better understand underlying cellular changes to tensor measures. Diffusivity measures of superficial WM in the temporal lobe frequently correlated with vascular size and glial density, suggesting these markers are responsive to vascular pathologies in epilepsy. Furthermore, inverse correlations of FA and myelin markers in temporal superficial WM were reported, highlighting the link between FA and demyelination of projecting WM fibres in superficial WM.^25^ Relating abnormalities to distance may subsequently help identify abnormalities near the potential epileptogenic zone in grey matter may be of use for lateralization and, potentially, localisation.

### WM abnormalities are similar between MRI-negative and MRI-positive HS

Our finding of superficial WM abnormalities, in patients considered MRI-negative, has potential clinical implementations for surgery. DWI is often acquired to map key white matter tracts to preserve during surgery, but is not typically used for lateralisation or localization. Mapping FA in superficial temporal WM may have utility for lateralisation and possibly support localisation. Indeed, several research studies have already hinted at this possibility using network-based techniques that leverage deep and superficial tracts,^26-30^ in addition to analyzing distances along tracts.^27,31^

One potential methodological limitation of this research was the influence of nearby hippocampal atrophy affecting nearby FA measures, possibly making reported abnormalities in FA a phenomenon of grey matter atrophy rather than superficial WM change. To investigate the influence of nearby GM atrophy; we compared to controls the three most superficial distance measures between the MRI-visible hippocampal sclerosis (MRI-positive), and the individuals with TLE with no MRI-visible pathology (MRI-negative). Differences between MRI-positive and negative TLE with controls were consistent at any of the superficial distances for FA measures. Thus, at the group level, superficial WM differences at these layers are not completely influenced by neighboring GM atrophy such as hippocampal sclerosis. Similar research reported no relationship between cortical thickness alterations and superficial WM DWI, but a negative correlation was reported between ipsilateral hippocampal volume and a multivariate diffusion tensor measures [combination of FA and mean diffusivity (MD)].^10^ Detecting abnormalities in both MRI-positive and MRI-negative TLE groups highlights the potential role this technique could provide in future lateralisation and even localisation.

### Differences in superficial WM are replicable

We analyzed two cohorts of drug-resistant TLE with differing DWI acquisitions: one used a high angular resolution multi-shell acquisition while the other used a single shell with 52 directions. Despite the differences in DWI parameters and image resolution, there were consistent findings in both cohorts. Therefore analyzing superficial WM holds promise for detecting abnormalities in standard clinical DWI data, indicating potential for future research.

### Superficial WM can lateralize epileptogenic tissue

Superficial WM was not only more abnormal than deep WM, but it also offered greater lateralization accuracy of 85% between epileptogenic ipsilateral and contralateral temporal lobe WM. Diffusion tensor imaging (DTI) measures have shown promise in the ability to correctly lateralize epileptogenic tissue. Pustina *et al.* (2015)^32^ investigated 58 drug-resistant TLE patients and found 71% accuracy using FA asymmetry measures to predict laterality. Though they reported other imaging types such as hypometabolism in PET to be more effective at lateralization, they only investigated deep WM tracts in the temporal lobe (fornix, parahippocampal, uncinate and inferior longitudinal fasciculi). Similar research showed 90% lateralization accuracy in 21 individuals with TLE, when comparing the FA values of all major WM tracts. Uncinate and parahippocampal gyri provided the greatest lateralization information.^33^ Only deep WM were analysed in these studies; it would be interesting to see if superficial WM could further increase lateralization accuracy. Studies have also investigated DTI measures of subcortical regions such as the hippocampus in a cohort of 23 unilateral TLE patients. Some DTI measures differed significantly between hemispheres, with RD showing the greatest lateralization out of the tensor measures with 73% accuracy.^34^ Future DTI research analyzing superficial and deep WM, in addition to cortical and subcortical grey matter regions at the individual level could therefore provide additional information for implantation and surgery decision-making in drug-resistant TLE.

### Wider implications

With superficial WM showing stronger DWI abnormalities, we also investigated whether superficial WM abnormality was associated with epilepsy duration. Although the first cohort did not show a significant correlation in either superficial or deep WM, the second cohort showed a weak relationship between WM abnormality and length of epilepsy. This effect was exclusive to the superficial and not the deep WM grouping, however (Supplementary Fig. A5). This is complementary to previous mixed findings suggesting WM abnormality is related to epilepsy duration.^35^ The relationship between epilepsy and specific WM depths (i.e. cognitive profiles, medication load, seizure severity) could help bring further understanding of the varying pathology in different epilepsies.

Though novel in epilepsy, analysing WM abnormalities specific to distance is more common in other neurological disorders such as Multiple Sclerosis (MS).^36,37^ DWI abnormalities in MS can be identified in visually normal WM and have been related to cortical lesion load.^36^ Studies in MS have reported that WM depth to the lateral ventricles affects the severity of WM damage, with greater WM damage nearer the ventricles.^37^ Understanding WM abnormalities relative to its depth in the brain could help heighten sensitivity for detecting abnormalities in epilepsy.

### Strengths and limitations

A strength of the current findings is the replication of these substantial differences in superficial white matter across two large TLE cohorts, despite significantly different DWI acquisitions. However, it must be noted that all individuals with TLE in these cohorts were drug-resistant. In light of previous studies reporting differences between drug-resistant and responsive TLE in DWI metrics at superficial WM distances.^11^ It is important to investigate if these findings are found in drug-responsive TLE. The current study captured all WM in the temporal lobe. It would be of interest for future studies to investigate superficial-versus-deep WM changes in cohorts with extratemporal epilepsies. Lastly, though we lost individual variability in distance from registering to a standard template, the subsequent WM mask and distance map provided a standardised distance allowing individuals to be directly compared. As abnormalities were related to that standardised distance in the current study, this may not reflect the exact location in that particular individual.

### Conclusion

DWI measures of FA show greater abnormalities at superficial than deeper WM depths in the temporal lobe of two different cohorts of drug-resistant TLE. The greatest superficial abnormality was evident within the ipsilateral temporal lobe. The differences were seen in both MR-positive (hippocampal sclerosis) and MR-negative patients, indicating that superficial WM abnormalities offer higher sensitivity and better lateralization, particularly 5 mm below the cortex. Superficial WM could be crucial for sensitive analysis, robust lateralisation, and can supplement traditional structural imaging.

## Supplementary Material

fcaf305_Supplementary_Data

## Data Availability

T1w scans and associated metadata are available as part of a previously published database.^38^ Diffusion-weighted scans will be made available in a future data release. Code for the analysis is available at the following location: https://github.com/cnnp-lab/SWM/.
