# Supplementary material for: Superficial and deep white matter abnormalities in temporal lobe epilepsy
Source: Brain Commun. 2025 Aug 19;7(5):fcaf305. doi: 10.1093/braincomms/fcaf305 (PMC12402771; doi:10.1093/braincomms/fcaf305)
Supplement: fcaf305_Supplementary_Data [file fcaf305_supplementary_data.docx]

Superficial and deep white matter abnormalities in temporal lobe epilepsy

Gerard R. Hall, Sarah J. Gascoigne, Jonathan J. Horsley, Yujiang Wang, Csaba Kozma, Jane de Tisi, Sjoerd B. Vos, Gavin P. Winston, John S. Duncan, Peter N. Taylor

# Appendices

## All additional post-hoc tests

Additional post-hoc tests from Section 3.1 are shown below. Comparisons between each of the distances is presented on Table A1, and the interactions between Hemisphere and Distance on Table A2.

|  |  |  |  |  | $\mu$ 95% Confidence Interval | |
| --- | --- | --- | --- | --- | --- | --- |
| Distance  Group 1 | Distance  Group 2 | $\mu$(mean difference) | $\mu$ standard error | p value | Lower bound | Upper bound |
| [1.0-2.0] | [2.0-3.0] | 0.19 | 0.05 | 0.01* | 0.03 | 0.34 |
| [1.0-2.0] | [3.0-4.1] | 0.09 | 0.09 | 0.91 | -0.16 | 0.34 |
| [1.0-2.0] | [4.1-5.1] | -0.23 | 0.11 | 0.22 | -0.53 | 0.07 |
| [1.0-2.0] | [5.1-6.1] | -0.34 | 0.13 | 0.10 | -0.72 | 0.04 |
| [1.0-2.0] | [6.1-7.1] | -0.68 | 0.13 | <.001* | -1.07 | -0.30 |
| [2.0-3.0] | [3.0-4.1] | -0.10 | 0.07 | 0.70 | -0.29 | 0.10 |
| [2.0-3.0] | [4.1-5.1] | -0.42 | 0.09 | <.001* | -0.69 | -0.14 |
| [2.0-3.0] | [5.1-6.1] | -0.53 | 0.12 | <.001* | -0.89 | -0.17 |
| [2.0-3.0] | [6.1-7.1] | -0.87 | 0.13 | <.001* | -1.25 | -0.49 |
| [3.0-4.1] | [4.1-5.1] | -0.32 | 0.05 | <.001* | -0.47 | -0.17 |
| [3.0-4.1] | [5.1-6.1] | -0.43 | 0.09 | <.001* | -0.69 | -0.17 |
| [3.0-4.1] | [6.1-7.1] | -0.77 | 0.11 | <.001* | -1.10 | -0.44 |
| [4.1-5.1] | [5.1-6.1] | -0.11 | 0.06 | 0.39 | -0.29 | 0.06 |
| [4.1-5.1] | [6.1-7.1] | -0.45 | 0.09 | <.001* | -0.71 | -0.19 |
| [5.1-6.1] | [6.1-7.1] | -0.34 | 0.07 | <.001* | -0.53 | -0.15 |

**Table A1: Post-hoc comparisons of the repeated measures ANOVA between the median FA from each of the 6 distance groups in cohort 1 (n=81).** All 6 distance groups are measured in millimeters (mm) as distance from GM surface. Confidence intervals are based of the mean.

|  |  |  |  |  | | $\mu$ 95% Confidence Interval | |
| --- | --- | --- | --- | --- | --- | --- | --- |
| Hemisphere | Distance  Group 1 | Distance  Group 2 | $\mu$(mean difference) | $\mu$standard error | p value | Lower bound | Upper bound |
| Ipsilateral | [1.0-2.0] | [2.0-3.0] | 0.195 | 0.070 | 0.07 | -0.008 | 0.400 |
| Contralateral | [1.0-2.0] | [2.0-3.0] | 0.176 | 0.061 | 0.056 | -0.003 | 0.355 |
| Ipsilateral | [1.0-2.0] | [3.0-4.1] | 0.033 | 0.105 | 1.00 | -0.273 | 0.338 |
| Contralateral | [1.0-2.0] | [3.0-4.1] | 0.146 | 0.095 | 0.646 | -0.132 | 0.424 |
| Ipsilateral | [1.0-2.0] | [4.1-5.1] | -0.373 | 0.113 | 0.02* | -0.704 | -0.042 |
| Contralateral | [1.0-2.0] | [4.1-5.1] | -0.087 | 0.122 | 0.980 | -0.442 | 0.269 |
| Ipsilateral | [1.0-2.0] | [5.1-6.1] | -0.606 | 0.139 | 0.001* | -1.013 | -0.199 |
| Contralateral | [1.0-2.0] | [5.1-6.1] | -0.081 | 0.155 | 0.995 | -0.533 | 0.371 |
| Ipsilateral | [1.0-2.0] | [6.1-7.1] | -0.939 | 0.158 | 0.001* | -1.400 | -0.478 |
| Contralateral | [1.0-2.0] | [6.1-7.1] | -0.428 | 0.148 | 0.055 | -0.861 | 0.005 |
| Ipsilateral | [2.0-3.0] | [3.0-4.1] | -0.163 | 0.078 | 0.299 | -0.389 | 0.064 |
| Contralateral | [2.0-3.0] | [3.0-4.1] | -0.031 | 0.077 | 0.999 | -0.257 | 0.195 |
| Ipsilateral | [2.0-3.0] | [4.1-5.1] | -0.568 | 0.100 | 0.001* | -0.857 | -0.279 |
| Contralateral | [2.0-3.0] | [4.1-5.1] | -0.263 | 0.111 | 0.180 | -0.587 | 0.061 |
| Ipsilateral | [2.0-3.0] | [5.1-6.1] | -0.801 | 0.135 | 0.001* | -1.196 | -0.407 |
| Contralateral | [2.0-3.0] | [5.1-6.1] | -0.263 | 0.144 | 0.482 | -0.680 | 0.164 |
| Ipsilateral | [2.0-3.0] | [6.1-7.1] | -1.134 | 0.161 | 0.001* | -1.603 | -0.665 |
| Contralateral | [2.0-3.0] | [6.1-7.1] | -0.604 | 0.137 | 0.001* | -1.004 | -0.204 |
| Ipsilateral | [3.0-4.1] | [4.1-5.1] | -0.405 | 0.066 | 0.001* | -0.597 | -0.214 |
| Contralateral | [3.0-4.1] | [4.1-5.1] | -0.232 | 0.064 | -0.007 | -0.421 | -0.044 |
| Ipsilateral | [3.0-4.1] | [5.1-6.1] | -0.639 | 0.110 | 0.001* | -0.961 | -0.317 |
| Contralateral | [3.0-4.1] | [5.1-6.1] | -0.227 | 0.106 | 0.279 | -0.537 | 0.083 |
| Ipsilateral | [3.0-4.1] | [6.1-7.1] | -0.971 | 0.143 | 0.001* | -1.390 | -0.553 |
| Contralateral | [3.0-4.1] | [6.1-7.1] | -0.574 | 0.125 | 0.001* | -0.939 | -0.208 |
| Ipsilateral | [4.1-5.1] | [5.1-6.1] | -0.233 | 0.074 | 0.026 | -0.448 | -0.018 |
| Contralateral | [4.1-5.1] | [5.1-6.1] | 0.005 | 0.075 | 1.000 | -0.213 | 0.223 |
| Ipsilateral | [4.1-5.1] | [6.1-7.1] | -0.566 | 0.112 | 0.001* | -0.894 | -0.238 |
| Contralateral | [4.1-5.1] | [6.1-7.1] | -0.342 | 0.106 | 0.021 | -0.650 | -0.033 |
| Ipsilateral | [5.1-6.1] | [6.1-7.1] | -0.333 | 0.085 | 0.002* | -0.580 | -0.085 |
| Contralateral | [5.1-6.1] | [6.1-7.1] | -0.347 | 0.08 | 0.001* | -0.589 | -0.105 |

**Table A2: Post-Hoc comparisons for the interaction between Hemisphere (ipsilateral, contralateral) and Distance groups (1-6) in the repeated measures ANOVA.** All 6 distance groups are measured in millimeters (mm) as distance from GM surface. Confidence intervals are based of the mean.

## Additional Patient Information and Analysis

**Summary of demographics and clinical variables**

|  | Cohort 1 | |  | Cohort 2 | |
| --- | --- | --- | --- | --- | --- |
|  | Patients | Controls |  | Patients | Controls |
| $N$ | 81 | 67 |  | 70 | 29 |
| Age $\left( M,SD \right)$ | 36.74, 10.98 | 40.27, 13.11 |  | 37.19, 10.88 | 37.66, 12.40 |
| Sex $\left( M,SD \right)$ | 36, 45 | 39, 42 |  | 37, 33 | 12, 17 |
| Lateralisation (L, R) | 44, 37 | - |  | 35, 35 | - |
| HS (Y, N) | 43, 38 | - |  | 33, 37 | - |
| MRI Visible (Y, N)  TLE type (Mesial, Lateral) | 74, 7  43, 38 | -  - |  | 57, 13  33, 37 | -  - |
| Onset Age $\left( M,SD \right)$ | 16.92, 11.34 | - |  | 17.08, 10.14 | - |
| Post-Op ILAE Y1 (1/2/3/4/5) | 38/11/8/10/3 | - |  | 36/9/8/13/4 | - |

## Table A3: Table summarising demographics and clinical variables of both cohorts. Age is calculated at scan. ILAE scores were not available for 11 patients in Cohort 1. Abbreviations: HS = Histologically confirmed Hippocampal sclerosis, MRI Visible = MRI confirmed hippocampal sclerosis. Post-Op ILAE Y1 = Count of international league against epilepsy scores after one year post operation (scores range between 1-5).

## Comparisons between MR Positive and Negative patients against controls

**First Cohort, Ipsilateral Temporal Lobe FA**

|  | MRI Positive HS ($n=41$) | | |  | MRI Negative ($n=7$) | | |
| --- | --- | --- | --- | --- | --- | --- | --- |
| WM Distance Grp | $t$ | $p$ | $d$ |  | $t$ | $p$ | $d$ |
| [1.0-2.0] | -7.268 | <.001* | -1.508 |  | -3.041 | 0.007* | -1.271 |
| [2.0-3.0] | -7.973 | <.001* | -1.702 |  | -2.795 | 0.013* | -1.154 |
| [3.0-4.1] | -6.937 | <.001* | -1.395 |  | -2.372 | 0.041* | -0.999 |
| [4.1-5.1] | -4.743 | <.001* | -0.945 |  | -1.733 | 0.175 | -0.722 |
| [5.1-6.1] | -2.772 | 0.013* | -0.530 |  | -2.057 | 0.087 | -0.777 |
| [6.1-7.1] | -1.220 | 0.450 | -0.242 |  | -1.329 | 0.376 | -0.495 |

## Table A4: Compared to controls, all paired t-tests ran per distance group for FA measures in the ipsilateral hemisphere comparing MR positive HS and negative patients against controls for the first cohort. All p-values were adjusted with a bonferroni correction for multiple comparison corrections between groups. Degrees of freedom was 106 for MRI positive and controls, and 72 MRI negative and control comparisons. Only TLE patients with hippocampal sclerosis were included in the MRI positive group. Abbreviations: t: t-statistic, d: Cohens d, p: Significance (p < .0.05 = ∗).

**Second Cohort, Ipsilateral Temporal Lobe FA**

|  | MR Positive HS ($n=33$) | | |  | MR Negative ($n=13$) | | |
| --- | --- | --- | --- | --- | --- | --- | --- |
| WM Distance Group | $t$ | $p$ | $d$ |  | $t$ | $p$ | $d$ |
| [1.0-2.0] | -7.028 | <.001* | -2.140 |  | -2.828 | 0.015* | -1.048 |
| [2.0-3.0] | -5.689 | <.001* | -2.104 |  | -4.107 | <.001* | -1.681 |
| [3.0-4.1] | -5.503 | <.001* | -1.952 |  | -5.106 | <.001* | -1.892 |
| [4.1-5.1] | -5.197 | <.001* | -1.674 |  | -5.063 | <.001* | -1.655 |
| [5.1-6.1] | -2.588 | 0.024* | -0.765 |  | -2.756 | 0.018 | -0.935 |
| [6.1-7.1] | -1.631 | 0.216 | -0.457 |  | -1.302 | 0.401 | -0.418 |

**Table A5: Compared to controls, all paired t-tests ran per distance group for FA measures in the ipsilateral hemisphere comparing MR positive HS and negative patients against controls for the second cohort.** All p-values were adjusted with a bonferroni correction for multiple comparison corrections between groups. Degrees of freedom was 60 for MR-positive and Controls, and 40 MR-negative and Control comparisons. Abbreviations: t: t-statistic, d: Cohens d, p: Significance (p < .0.05 = ∗).

##
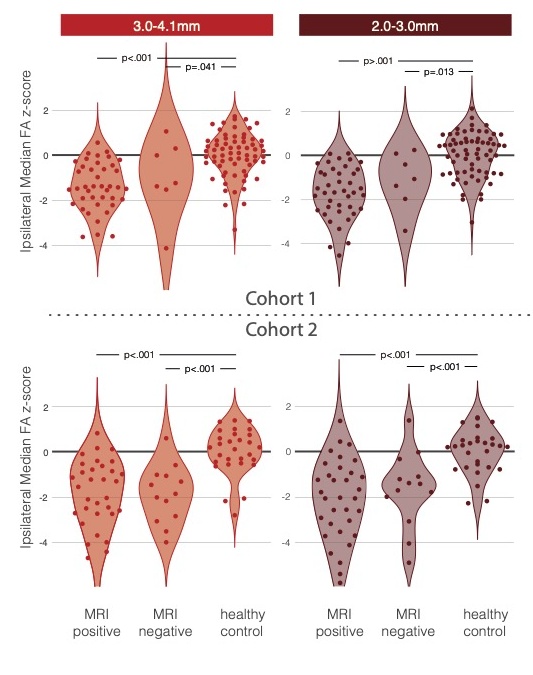
Comparison figures for additional superficial distance groupings in both cohorts

## Figure A1: Similar group abnormalities were present for both MRI positive-HS and MRI-negative individuals against controls at the most superficial WM distances for both cohorts. For each distance grouping in cohort 1 and cohort 2, each datapoint represents an individual with either: MR-positive TLE (cohort 1: n=74, cohort 2: n=57), MR-negative TLE (cohort 1: n=7, cohort 2: n=13) or healthy control (cohort 1: n=67, cohort 2: n=29). Suggesting the differences seen at the superficial level are not from neighbouring GM atrophy. Only patients exclusively with hippocampal sclerosis were included in the MRI positive group. Significance tests were independent samples t-tests. Abbreviations: WM: white matter


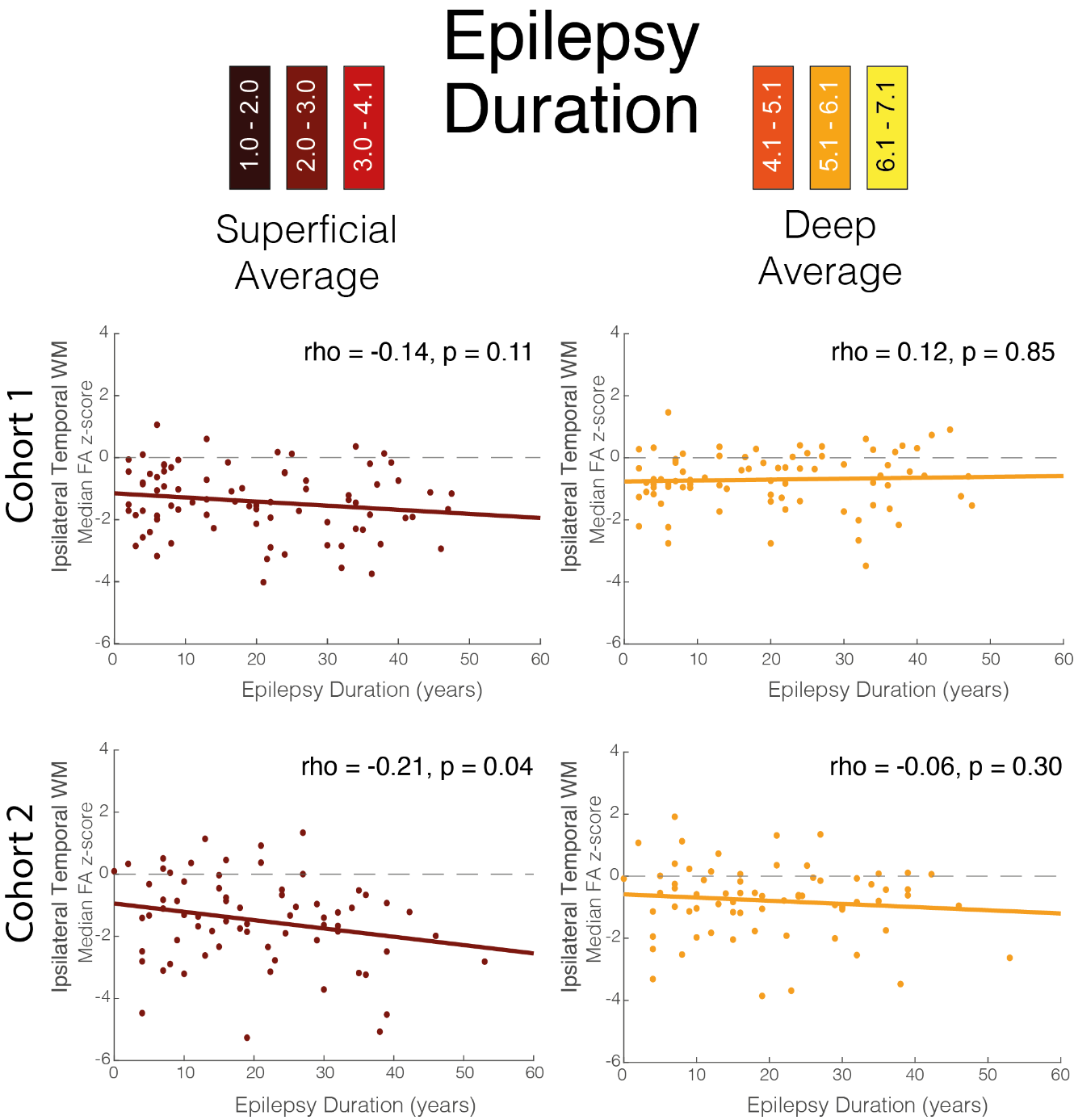
**Ipsilateral temporal superficial and deep WM correlations with length of epilepsy**

**Figure A2. Scatter plots illustrating the relationship between epilepsy duration in both cohorts (cohort 1 (upper): n=81, cohort 2 (lower): n=70) and the average ipsilateral median FA for the three most superficial (left) and deep temporal WM distances (right).** Correlational analysis was ran using a one-tailed spearman’s rho, only the average superficial distance of cohort 2 (lower, left scatterplot) had a significant weak negative correlation with duration of epilepsy (rho = -0.21, p = 0.04). In contrast, the average deep distance of ipsilateral temporal WM in cohort 2 (lower, right scatterplot) was not significant (rho = -0.06, p = 0.30). Both superficial and deep (upper, left scatterplot and upper, right scatterplot respectively) were non-significant for cohort 1 however (superficial: rho = -0.14, p = 0.11; deep: rho = 0.12, p = 0.85). Lines of best fit in each scatterplot are shown for representative purposes only. Abbreviations: FA: fractional anisotropy, WM: white matter.

**
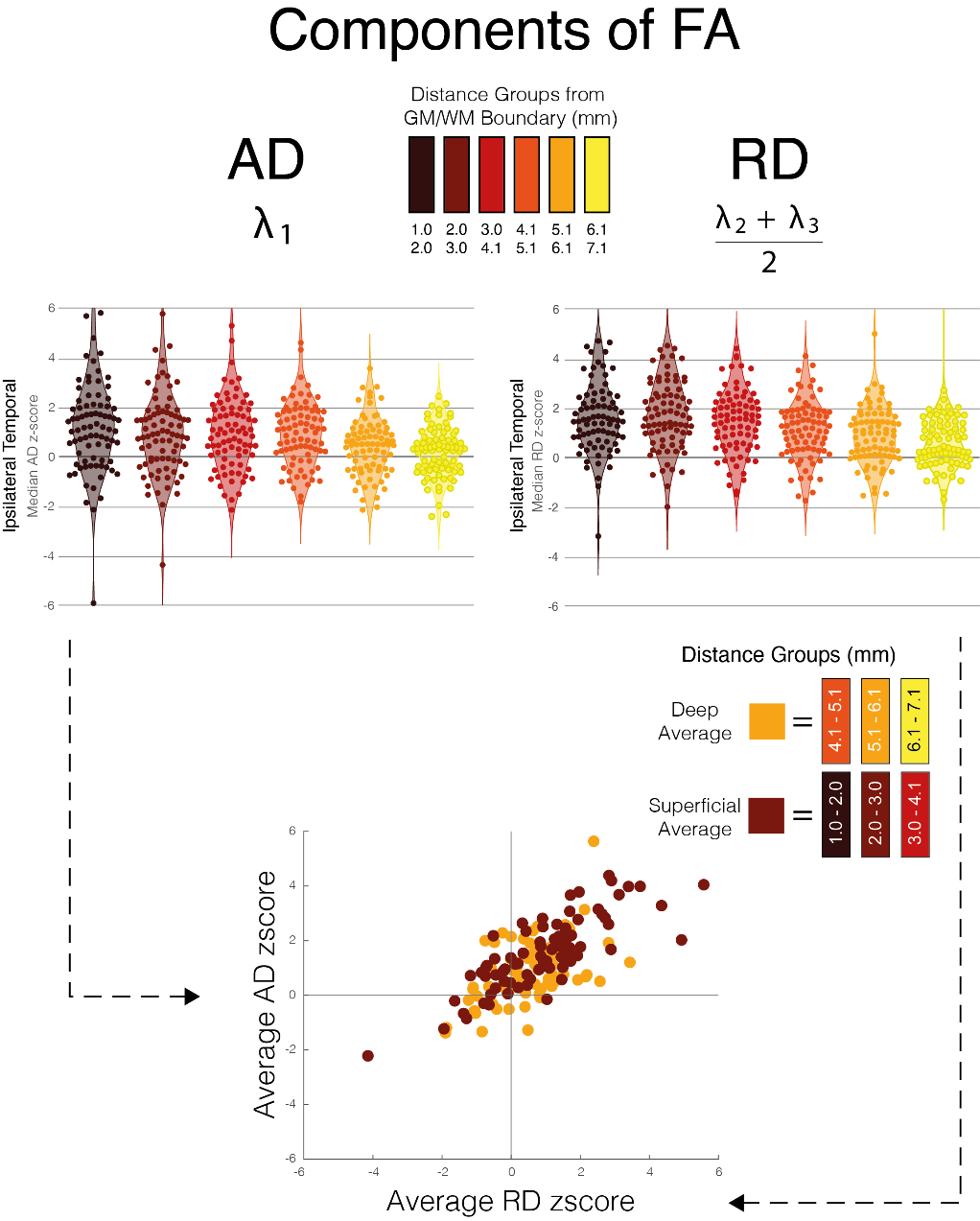
Breakdown of the component measures that make up FA**

**Figure A3. Shows the underlying measures of FA; AD and RD in the ipsilateral temporal WM at each distance for cohort 1 (n=81).** AD and RD measures underwent the same processing pipeline as FA. (Upper) Each datapoint represents an individual with TLE (n=81) for each of their distance groupings for AD (left) and RD (right) measures. (Lower) The three most superficial [1.0mm – 4.1mm] and deep [4.1mm – 7.1mm] distance groups have been averaged together. Each datapoint again represents an individual with TLE (n=81) but with superficial and deep averages overlaid and plot in relation to average AD and RD z-score. Both AD and RD measures show greater increase in z-scores at the superficial distances compared to deeper temporal WM distances. From visual inspection in relation to AD, each corresponding RD measure showed an even greater z-score increase, driving this reduction in FA. Therefore, with both AD and RD z-scores being greatly increased at the superficial distances, measures of MD would therefore show similar abnormal increases at superficial distances. Abbreviations: FA: fractional anisotropy, AD: axial diffusivity, RD: radial diffusivity, WM: white matter MD: mean diffusivity.

**
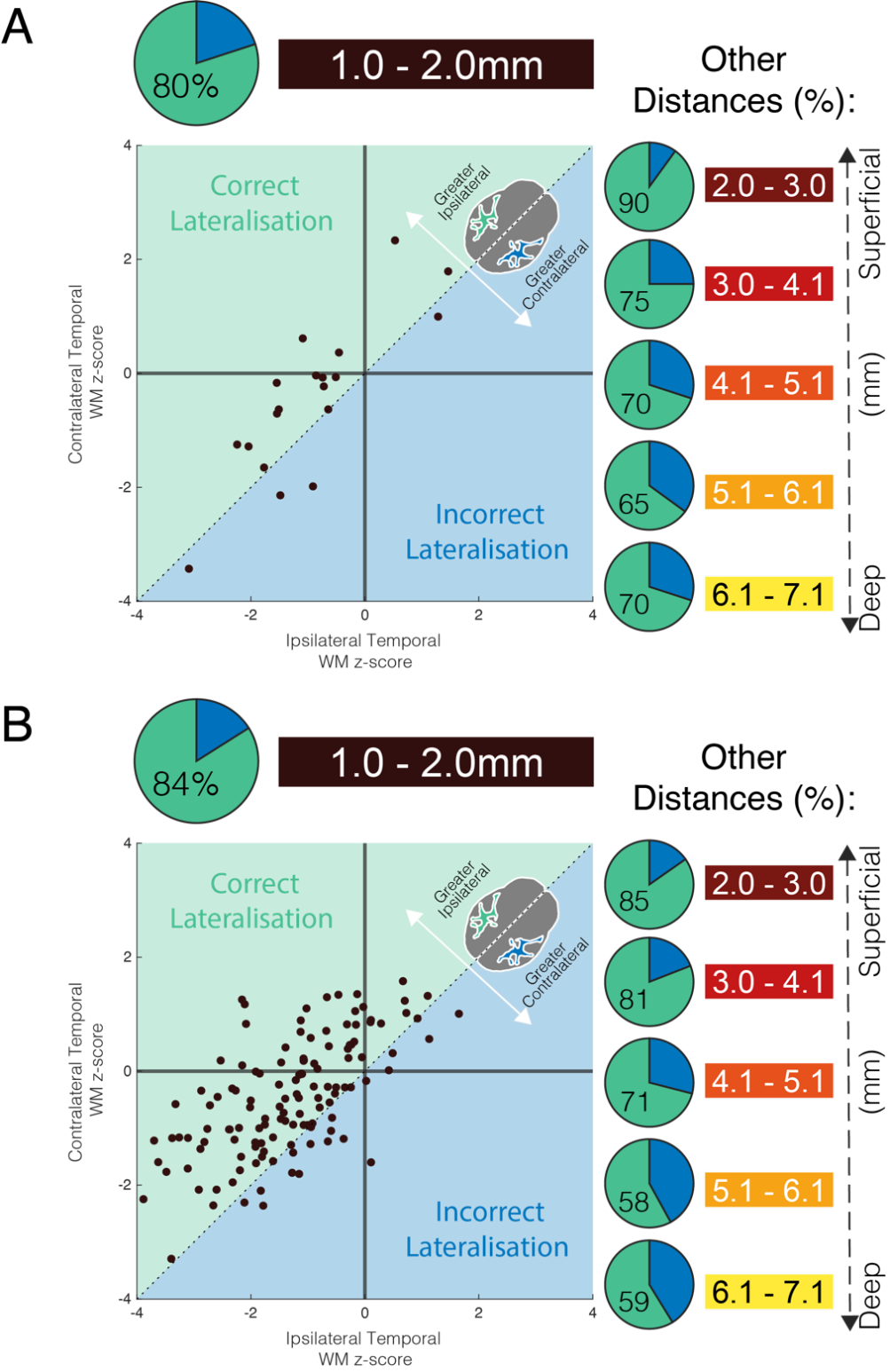
Lateralisation effectiveness of superficial WM in MRI positive and negative patients**

**Figure A4. Ability to lateralise epileptogenic tissue in superficial WM is similar in MRI negative (A) and positive TLE patients (B).** Replicating Figure 5, each datapoint represents an individual with TLE in cohort 1: (A) MRI negative (n=7) and (B) MRI positive (n=74) patients have been separated to help illustrate lateralisation based on largest superficial WM temporal z-score is consistent in both groups.


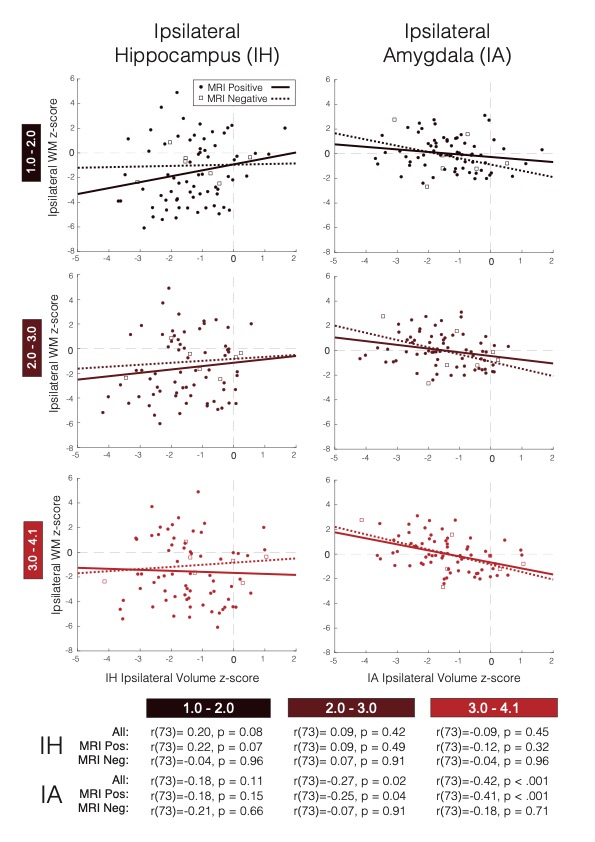
**Relationship between ipsilateral WM z-score and the volume of the ipsilateral hippocampus and amygdala**

**Figure A5. Cohort 1 (n=75): Relationship between Ipsilateral WM z-score for MRI positive and negative TLE at the three most superficial WM depths and the z-score of the volume in the ipsilateral hippocampus (IH) and ipsilateral amygdala (IA).** Each datapoint in the scatterplots represent an individual with MRI positive or negative TLE from cohort 1. Correlational analysis was ran using a two-tailed spearman’s rho. All volumes were calculated from the each T1w scan, using FreeSurfer. Six individuals with TLE did not pass quality control from the original 81 of cohort 1 due to poor segmentation of the hippocampus and amygdala.


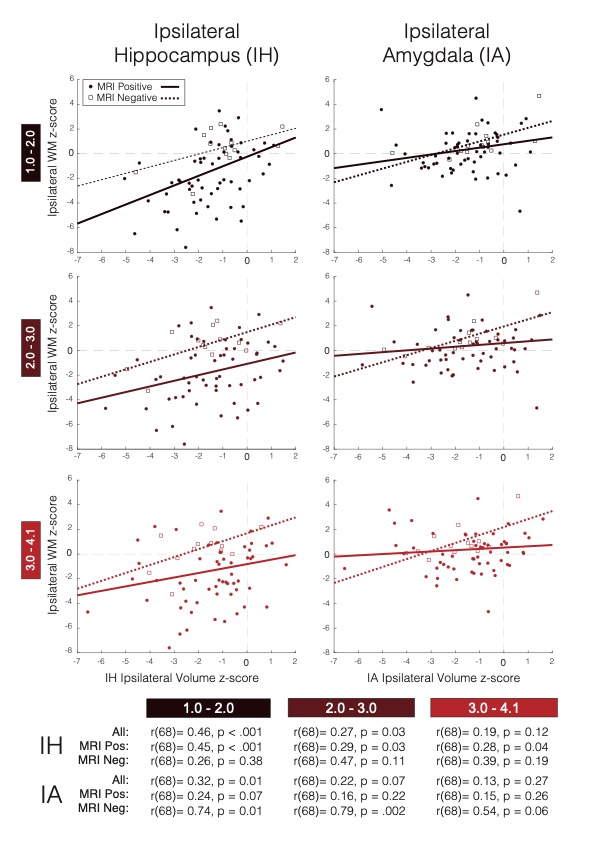


**Figure A6. Cohort 2 (n=70): Relationship between Ipsilateral WM z-score for MRI positive and negative TLE at the three most superficial WM depths and the z-score of the volume in the ipsilateral hippocampus (IH) and ipsilateral amygdala (IA).** Each datapoint in the scatterplot represent an individual with MRI positive or negative TLE from cohort 2. Correlational analysis was ran using a two-tailed spearman’s rho. All volumes were calculated from each individual corresponding T1w scan, using FreeSurfer.

**Differences in FA z-scores between lateral and mesial TLE at all WM distance groups**

|  |  | |  |  | |  |  | | | |
| --- | --- | --- | --- | --- | --- | --- | --- | --- | --- | --- |
| WM Distance  Group | Lateral TLE | |  | Mesial TLE | |  | Comparisons (t-test) | | | |
|  | $\mu$ | SD |  | $\mu$ | SD |  | *df* | *t* | *p* | *d* |
|  |  |  |  |  |  |  |  |  |  |  |
| 1.0-2.0 | -1.19 | 1.12 |  | -1.48 | 1.12 |  | 79 | -1.14 | 0.26 | -0.28 |
| 2.0-3.0 | -1.41 | 1.07 |  | -1.65 | 1.23 |  | 79 | -0.92 | 0.36 | -0.24 |
| 3.0-4.1 | -1.38 | 1.26 |  | -1.37 | 1.18 |  | 79 | 0.03 | 0.98 | 0.01 |
| 4.1-5.1 | -1.05 | 1.11 |  | -0.90 | 1.03 |  | 79 | 0.61 | 0.55 | 0.14 |
| 5.1-6.1 | -0.95 | 1.00 |  | -0.55 | 0.96 |  | 79 | 1.86 | 0.07 | 0.40 |
| 6.1-7.1 | -0.48 | 1.00 |  | -0.34 | 1.03 |  | 79 | 0.61 | 0.54 | 0.14 |

**Table A6. Differences in lateral and mesial TLE patients in cohort 1 (n=83) for all WM distance groups.** Values are specific to z-scores calculated from controls using the median FA measures per distance. Abbreviations: $\mu$ : Mean, SD: Standard deviation, t: t-statistic, d: Cohens d, p: Significance (p < .0.05 = ∗).

|  |  | |  |  | |  |  | | | |
| --- | --- | --- | --- | --- | --- | --- | --- | --- | --- | --- |
| WM Distance  Group | Lateral TLE | |  | Mesial TLE | |  | Comparisons (t-test) | | | |
|  | $\mu$ | SD |  | $\mu$ | SD |  | *df* | *t* | *p* | *d* |
|  |  |  |  |  |  |  |  |  |  |  |
| 1.0-2.0 | -0.96 | 1.40 |  | -1.94 | 1.26 |  | 68 | -3.06 | .003* | -0.98 |
| 2.0-3.0 | -1.13 | 1.55 |  | -1.97 | 1.63 |  | 68 | -2.20 | 0.03* | -0.84 |
| 3.0-4.1 | -1.26 | 1.51 |  | -1.76 | 1.63 |  | 68 | -1.34 | 0.19 | -0.50 |
| 4.1-5.1 | -1.14 | 1.42 |  | -1.54 | 1.44 |  | 68 | -1.16 | 0.25 | -0.40 |
| 5.1-6.1 | -0.57 | 1.24 |  | -0.73 | 1.18 |  | 68 | -0.55 | 0.58 | -0.16 |
| 6.1-7.1 | -0.36 | 1.22 |  | -0.44 | 1.20 |  | 68 | -0.30 | 0.76 | -0.09 |

**Table A7. Differences in lateral and mesial TLE patients in cohort 2 (n=70) for all WM distance groups.** Values are specific to z-scores calculated from controls using the median FA measures per distance. Abbreviations: $\mu$ : Mean, SD: Standard deviation, t: t-statistic, d: Cohens d, p: Significance (p < .0.05 = ∗).

**Comparisons between MR-positive and negative TLE patients in both cohorts at the deeper WM distance groups**

**
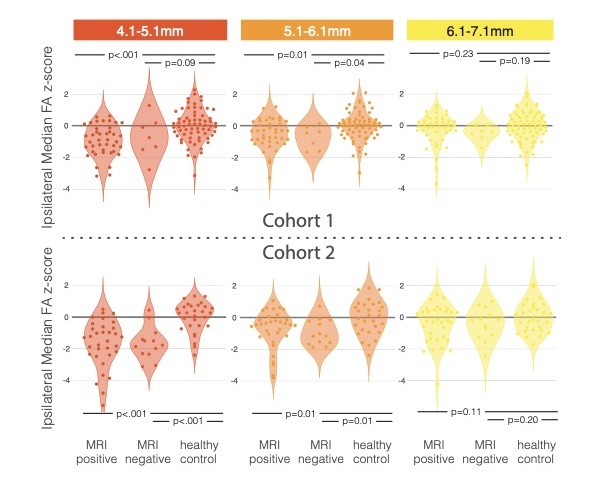
**

**Figure A7. Similar group abnormalities were present for both MRI positive-HS and MRI-negative individuals against controls at the deepest WM distances for both cohorts.** Each datapoint in the grouped scatterplots represents an individual at a specific distance grouping. Cohort 1: MRI-positive (n=74), MRI-Negative (n=7), Healthy control (n=67). Cohort 2: MRI-positive (n=57), MRI-Negative (n=13), Healthy control (n=29). Significance tests were independent samples t-tests. Abbreviation: WM: white matter.
